# Obesity induced alterations in redox homeostasis and oxidative stress are present from an early age

**DOI:** 10.1371/journal.pone.0191547

**Published:** 2018-01-25

**Authors:** Alfonso M. Lechuga-Sancho, David Gallego-Andujar, Pablo Ruiz-Ocaña, Francisco M. Visiedo, Ana Saez-Benito, Mónica Schwarz, Carmen Segundo, Rosa M. Mateos

**Affiliations:** 1 Research Unit, Puerta del Mar University Hospital, Cadiz, Spain; 2 Pediatrics Department, Puerta del Mar University Hospital, Cádiz, Spain; 3 Department of Child and Mother Health and Radiology, Cadiz University Medical School, Cadiz, Spain; 4 Clinical Analysis Department, Puerta del Mar University Hospital, Cádiz, Spain; 5 “Salus Infirmorum” Faculty of Nursing. Cadiz University, Cadiz, Spain; 6 Department of Biotechnology, Biomedicine and Public Health, Cadiz University Medical School, Cadiz, Spain; Universitat de Lleida-IRBLLEIDA, SPAIN

## Abstract

**Objectives:**

Oxidative stress and inflammation have been postulated as underlying mechanisms for the development of obesity-related insulin resistance. This association however, remains elusive especially in childhood. We sought to investigate this relation by measuring oxidative stress and antioxidant response biomarkers, before and during an oral glucose tolerance test (OGTT), in different biological samples from obese children.

**Subjects:**

24 children were recruited for the study, (18 obese and 6 controls). After OGTT, the obese group was subdivided in two, according to whether or not carbohydrate metabolic impairment (Ob.IR+, Ob.IR-; respectively) was found. Different biomarkers were analyzed after fasting (T = 0) and during an OGTT (T = 60 and 120 min). Lipoperoxides were measured in plasma, erythrocytes, and urine; while advanced glycation end products were determined in plasma, and redox status (GSH/GSSG ratio) in erythrocytes.

**Results:**

We found marked differences in the characterization of the oxidative status in urine and erythrocytes, and in the dynamics of the antioxidant response during OGTT. Specifically, Ob.IR+ children show increased oxidative stress, deficient antioxidant response and a significant imbalance in redox status, in comparison to controls and Ob.IR- children.

**Conclusion:**

Obese children with insulin resistance show increased levels of oxidative stress biomarkers, and a stunted antioxidant response to an OGTT leading to increased oxidative stress after a single glucose load, as detected in erythrocytes, but not in plasma. We propose erythrocytes as sensors of early and acute changes in oxidative stress associated with insulin resistance in childhood obesity. This is a pilot study, performed with a limited sample size, so data should be interpreted with caution until reproduced.

## Introduction

Oxidative stress is one of the main molecular mechanisms involved in the development of obesity associated complications. Indeed, multiple biomarkers of oxidative stress are being identified as predictors of diabetes and cardiovascular events [1–4]. Results from different groups analysing oxidative stress in blood and urine in obese adults are controversial [5–7], denoting that not all obese individuals respond with the same antioxidant capacity to similar environmental stressors. Some have suggested this may vary according to the degree of the disease development and/or severity [8]. Nevertheless, most published studies measure these biomarkers in basal conditions [9,10], but their dynamics after a stressor, such as a glucose overload (OGTT), and more specifically, at pediatric age has not been explored yet.

Advanced glycation end products (AGEs) are also relevant contributors to insulin resistance in adult patients [11]. AGEs are target molecules with high capacity to activate the ubiquitous “Plasma Membrane Redox System” (PMRS), which transfers electrons from intracellular donors (NADPH, ascorbate) to extracellular acceptors. This PMRS’ action is linked to the generation of superoxide radicals and other reactive oxygen/nitrogen species (ROS/RNS) by NADPH oxidase. Physiologically, basal ROS work as second messengers, activating the expression of antioxidant genes. However, in the presence of insulin resistance and/or diabetes, ROS is produced in excess, inducing an inflammatory response in the vascular wall [12]. Consequently, increased AGEs may lead to an impaired redox balance, contributing to the pathogenesis of insulin resistance in obese adults.

Erythrocytes (RBCs), being highly specialized cells in the organism, function as a cell oxygen conveyor. Their unique structural features (lack of nuclei, mitochondria and ribosomes), make them highly sensitive to oxidative changes [13]. Erythrocyte’s antioxidant systems are well characterized [14] and redox imbalance in the erythrocyte has been previously demonstrated in response to metabolic complications [15–17].

Given the lack of consistency in the literature on oxidative stress plasmatic markers in obese individuals, we sought to investigate their levels in different biological samples of healthy and obese children. Two different settings were chosen: (i) basal conditions and (ii) an obese group during an OGTT, subdivided according to the presence or absence of metabolic complications.

We analysed various oxidative stress biomarkers in urine, plasma and RBCs in the three groups, finding differences in urine and RBCs in obese children with insulin resistance. Furthermore, we demonstrated that this group also display altered dynamics in their antioxidant response to OGTT as measured in RBCs.

## Subjects and methods

### Study design

Prospective and descriptive clinical study, designed to analyse the differences in oxidative stress biomarkers in response to an OGTT according to the glucose metabolism status in a group of obese children.

### Study subjects

The study was performed on a group of obese children of both sexes, with a control group for the basal condition (total N = 24) aged between 4 and 14 (Table 1). The non-obese group consisted of healthy children who needed a blood test, in the context of health controls (controls; N = 6), while the obese group (N = 18) was recruited among patients attending the Pediatric Endocrinology Unit of our centre, who needed an OGTT to assess their carbohydrate metabolism. Inclusion criteria were children between 4 and 14 years, with a BMI > +2 SD [18], and personal or family risk factors for metabolic complications (i.e. family history of hypertension, cardiovascular disease or diabetes mellitus in first grade relatives aged < 55 years, personal history of having been born small for gestational age, or big for gestational age from a mother with gestational diabetes, as well as presenting acanthosis nigricans). Patients whose obesity had a known or suspected organic or genetic cause, as well as patients with psychopathologies were not eligible for the study. All of the obese patients had sedentary habits (i.e. more than 2 hours/day of “screen activities”), and none of them engaged in structured physical activity more than 3 times per week. For the three days prior to the OGTT, patients were all asked to avoid performing any strenuous physical activity, and to keep a balanced diet, containing at least 150 g of carbohydrate per day. Finally, they were also asked not to eat, drink or exercise for at least 8 hours before the programmed OGTT.

**Table 1:** Characteristics of study population.

	CONTROL	Ob. IR-	Ob. IR+
**Anthropometric parameters**	* *	* *	* *
Number of subjects (N)	6	6	12
Sex (n° of boys)	4	4	4
Age (years)	10.3 ± 0.8	11.1 ± 1.2	10 ± 0.7
Tanner stage			
-Tanner I	6	2	8
-Tanner II-V	0	4	4
Height (cm)	135.2 ± 4.79	133.9 ± 23.2	141.2 ± 15.71
Height (SD)	-1.30±0.6	0.91 ± 0.9	0.75 ±0.2
Waist Cincumference (cm)	60.8 ± 1.8	91 ± 12.7ª	96.10 ± 7.6ª
Weight (kg)	30.8 ± 2.3	78.9 ± 8.3ª	64.9 ± 5.3ª
Weight (SD)	-0.88± 0.3	4.26± 0.8ª	4.26± 0.8ª
BMI (Kg /m2)	16.9± 0.9	33.9 ± 2.2ª	30 ± 1ª
BMI (SD)	-0.53 ± 0.3	4.2 ± 0.7ª	3.6 ± 0.3ª
Gestational age (weeks)	39 ± 0.9	40.3 ± 0.7	39.5 ± 0.7
Newborn weight (g)	2816 ± 51.4	3300 ±187.7ª	3430 ± 163,7ª
Newborn weight (SD)	-0.88± 0.2	-0.08± 0.5	0.71± 0.3
Newborn length (cm)	48 ± 0.5	52 ± 1.3	50 ± 0.9
Newborn length (SD)	-0.79± 0.2	1.29± 0.6	0.77± 0.2
Systolic Blood Pressure	94.25 ± 1.5	107.8 ± 13.5	122.4 ± 16.21ª
Diastolic Blood Pressure	63.75 ± 1.84	69 ± 8.2	65.83 ± 10.65
**Biochemical parameters**			
Blood glucose curve (mg/dL)*			
0 min	83 ± 1.8	88.00 ± 8.6	90.50 ± 6.70
30 min	n.d.	135.3 ± 6.8	139.4± 7.4
60 min	n.d.	111.6 ± 8.6	127.8 ± 5.7 ^b^
90 min	n.d.	114.3 ± 7.6	116.2 ± 7.1^b^
120 min	n.d.	113 ± 8.9	116.1 ± 5.8
Blood Insulin curve (mUI/mL)*			
0 min	5 ± 0.4	8.7 ± 3.5	16.4 ± 3.6^b^
30 min	n.d.	73.9 ± 27.1	128.1 ±38.1
60 min	n.d.	48.79 ± 11.3	122.2 ± 26.4
90 min	n.d.	31.46 ± 8.1	108.8 ± 25.4^b^
120 min	n.d.	30.12 ± 6.2	111.1 ± 24.8^b^
Total Cholesterol (mg/dL)	166.6 ± 9.1	150.5 ± 6	167 ± 8
Triglycerides (mg/dL)	41.3 ± 1.4	67.3 ± 11.9ª	84 ± 8.3 ª
HDL-C (mg/dL)	78.3 ± 4.2	55.2± 1.8ª	52 ± 3.3ª
LDL-C (mg/dL)	102.6 ± 6.1	96.4 ± 5.7	98.6 ± 6.9
Creatinine	0.5 ± 0.1	0.5 ± 0.1	0.53 ± 0.07
GOT (U/L)	23 ± 0.61	25.8 ±1.2	26.3 ±1.4
GPT(U/L)	14 ± 0.6	19.4 ± 1.0	28.4 ± 2.1ª
HOMA-IR	1.02 ± 0.1	1.6 ± 0.9	4.5 ± 1.0^ ab^
HOMA-Iβ	85 ± 5	128 ± 27	261 ± 61^ab^
**Pro-inflammatory cytokines**			
CRP (pg/mL)	0.4 ± 0.1	2.3 ± 0.9ª	4.1 ± 0.8^ ab^
IL-6 (pg/mL)	1.8 ± 0.5	4.1 ± 1.3ª	2.2 ± 0.5
TNF-α (pg/mL)	3.4 ± 0.5	4 ± 0.6	4.6 ± 0.5
Leptin (μg/mL)	3.6 ± 1.1	23.7± 4.8ª	38.6 ± 3.6^ab^
HGF(pg/mL)	118.1 ±19.9	172.8 ± 20.5	155.5 ± 18.1

Values are means ± SEM for 6 or 12 participants, depending of the group into the study population. (*) is indicative of glucose and insulin levels, respectively, before (0 min) and along 75 gr. OGTT. P<0.05 was considered for statistical significance, relative to control at baseline (a) and relative to Ob.IR- (b).

The obese group was later subdivided according to their carbohydrate metabolism characterization into a group with no evidence of carbohydrate metabolic impairment (Ob.IR-) and a group presenting at least one of them (Ob.IR+), (i.e. Impaired fasting glucose (IFG), impaired glucose tolerance (IGT) and insulin resistance (IR)—considered when HOMA-IR ≥ 3.5 as described Garcia-Cuartero *et al* 2007 [19]). None of our patients met the diagnostic criteria for diabetes mellitus.

The OGTT was performed in the clinic after overnight fasting, with 75 g of glucose (GlycoSull, QCA S.A., Tarragona, Spain). Healthy children serving as controls were not subjected to the OGTT. The study was carried out in accordance with *The Code of Ethics of the World Medical Association (Declaration of Helsinki) for experiments involving humans* and it was approved by the Human Ethics Committee of University Hospital Puerta del Mar (Cadiz, Spain). Written informed consent was obtained from each participant/parents.

### Sample collection and preparation

Venous blood samples were extracted in serum-separating tubes from every child after overnight fast (T0), and at 30, 60, 90 and 120 minutes during the OGTT in obese children. Extra samples in EDTA-coated tube were withdrawn at 0, 60 and 120 min for the immediate purification of total plasma and RBC for the oxidative stress and antioxidant biomarkers determination. Additionally, a single urine sample was obtained from every subject on the same day, before the OGTT for 8-isoprostane determination.

Each sample of total venous blood in EDTA-coated tubes was centrifuged 1600 x g at 4°C for 10 minutes to separate plasma (as supernatant) and erythrocytes (S2 Appendix). The upper layer of the red blood cell pellet containing the buffy coat was removed and erythrocytes were treated as follows. First, erythrocytes were washed 3 times in cold saline (0.9% NaCl) and then lysated by hypotonic shock with (1:40; v/v) distilled water. A supplementary step of treatment with (1:2; v/v) Chloroform: Ethanol was performed to denature haemoglobin, present in a very high concentration compared to other proteins in erythrocytes. The debris was separated from the intraerythrocytary fraction (RBC fraction from now on), by centrifugation.

### Variables

#### Anthropometry and biochemical analysis of the study population

The treating physician would perform a complete medical history and a full physical examination, registering the following data; age, gender, family history of dyslipemia, cardiovascular disease, obesity hypertension or diabetes, patients’ neonatal data such as gestational complications, gestation duration, and neonatal weight, length and cranial circumference, patients’ present height in cm using a fixed wall stadiometer, weight in a SECA 5000 balance, and used these to calculate the Body Mass Index (BMI), abdominal circumference, Tanner stage, blood pressure with appropriate arm pieces and the presence or absence of acantosis nigricans. Every anthropometrical variable was registered also in standard deviations for age and gender, using Spanish reference values. A summary of these variables are listed in Table 1.

The biochemical characterization included a lipid profile with total Cholesterol, HDL-Col, LDL-Col and TGs levels. Basal glucose and insulin were measured in every subject, while only the obese group were challenged with the OGTT, as detailed above. Furthermore, we calculated the ‘homeostasis model assessment of insulin resistance’ (HOMA-IR) by applying the formula: HOMA-IR = [(fasting insulin (mIU/L) x fasting glucose (mg/dl))/405)], and the homeostasis model assessment pancreatic β-cell function (HOMA-Iβ) was calculated using the following formula: HOMA-Iβ = [(fasting insulin (mIU/L) x 360) / (fasting glucose (mg/dl)-63)]. Biochemical analysis was determined in serum by using standard clinical assays at the Clinical Analysis Department, of our Hospital. Insulin was measured in a Roche Analytics E-170 analyzer using the ‘INSULIN 100 TESTS/KT’ (Roche Diagnostics, Indianapolis, IN, USA) [imprecision insulin assay was 2,26%], and serum glucose was analysed by the hexokinase method in a C8000 analyzer (Roche Diagnostics, Indianapolis, IN, USA) [imprecision glucose assay was 2% at standard level (100 mg/dl) and 3% at top level (240 mg/dl)] [20].

#### Pro-inflammatory cytokines

Basal levels of C-reactive protein (CRP), tumor necrosis factor-α (TNF-α), interleukin-6 (IL-6), leptin and hepatic growth factor (HGF) were measured in serum by multiplex immunoassay, using the ‘Milliplex Map Kit. Human adipokine magnetic bead panel 2’ (EMD Millipore Corporation, Billerica, MA, USA), following manufacturer’s instructions and quantified using a ‘Luminex 100/200^TM^ IS’.

#### Advanced Glycation End Products

(AGEs) were analysed in plasma using the ‘OxiSelect^TM^ AGE Competition Elisa Kit’ (Cell Biolabs, San Diego, CA, USA), following manufacturer’s instructions. Data were expressed as μg of carboxymethyl-lysine (CML) per millilitre of plasma, CML being the most prevalent AGE *in vivo*.

#### Oxidative stress markers

Lipid peroxidation. Thiobarbituric acid-reacting substances (TBARS) were measured by the spectrophotometric method described by Buege [21]. Results were expressed as nmol of MDA equivalents per mL of plasma or nmol of MDA per mg of proteins in RBCs. As a sign of oxidative damage, this is our main variable, and its increased levels in plasma and/or erythrocytes with the OGTT was our primary outcome measure.

8-isoprostane (8-iso PGF_2α_). Urine samples were tested for *8-iso PGF*_*2α*_ by the enzyme immunoassay analysis, ‘8-Isoprostane Express EIA Kit’ (Cayman Chemical Company, Ann Arbor, MI, USA), following manufacturer’s instructions. Data were expressed as pg of 8-iso PF_2α_ per mg creatinine.

#### Antioxidant activity markers

Total antioxidant capacity assay (TAC) was measured by the method described by Erel [22]. Results were expressed as μmol of Trolox equivalents per mL of plasma or per mg of proteins in the RBC fraction.

Determination of total erythrocyte glutathione equivalents (tGSH) were performed following the ‘DTNB-GSSG Reductase Recycling Assay for GSH and GSSG’[23]. It consists of monitoring the rate of 5-thio-2-nitrobenzoic acid (TNB) formation as result of GSH oxidation by the 5,5’-dithiobis (2-nitrobenzoic acid) (DTNB). The resulting GSSG is reduced to GSH by the action of the highly specific glutathione reductase and NADPH. TNB formation is monitored at 412 nm, being proportional to the sum of GSH and GSSG present in the sample. Additionally, GSSG concentration can be analysed by the same method if samples are previously derivatized with N-ethylmaleimide (NEM) and 2-vinylpyridine. Finally, redox status was expressed as GSH/GSSG ratio.

Catalase activity (CAT) was determined spectrophotometrically following hydrogen peroxide breakdown at 240 nm, as described by Aebi [24].

#### Statistical analysis

The significance tests used were the Mann–Whitney U test for pairwise comparisons, and Kruskal-Wallis non-parametric test followed by the Dunn's test, for comparisons of more than two groups. The calculated power of our sample size for inter-group comparison, to see a clinically relevant effect, (> 20% of controls’) [25] is 90.4%. We calculated this power using EPIDAT 4.2 software (available at https://www.sergas.es/Saude-publica/EPIDAT-4-2). A p<0.05 was accepted as a significant difference between groups.

## Results

### Anthropometrical and biochemical characterization of study population

As shown in Table 1, age was similar in every group, though there were differences in Tanner Stage, since children in the control group were all Tanner I. Conversely, there was a different proportion of Tanner ≥ II in the obese groups. There were also obvious differences in obesity-related parameters such as waist circumference and BMI (SD) between non-obese and obese children. Those differences were independent of sex (results not shown).

As expected by the definition of the study groups, there were significantly higher glucose and insulin levels along the OGTT, and elevated HOMA-IR and HOMA-Iβ indexes, in the Ob.IR+ group. Additionally, a significant increase in systolic blood pressure was detected in Ob.IR+ as compared to Ob.IR- and controls. None of our patients were diabetic, only one had IFG (basal glucose = 103 mg/dl) and 2 had IGT, (glucose at min 30 = 166 and 155 respectively, and both normalized at min 60), most of them being solely insulin resistant.

The Ob.IR+ group was found to have higher levels of Glutamyl pyruvic transaminase (GPT), and elevated levels in venous blood of pro-inflammatory cytokines such as CRP, IL6, TNF-α, Leptin and HGF, in contrast to healthy children. Ultrasonography was performed in every case in which transaminases were increased, and findings suggestive of non-alcoholic steatohepatitis were found in all but two of these patients.

### Oxidative stress markers in blood and urine of healthy and obese children. Effects of OGTT in both obese groups

**Fig 1:**
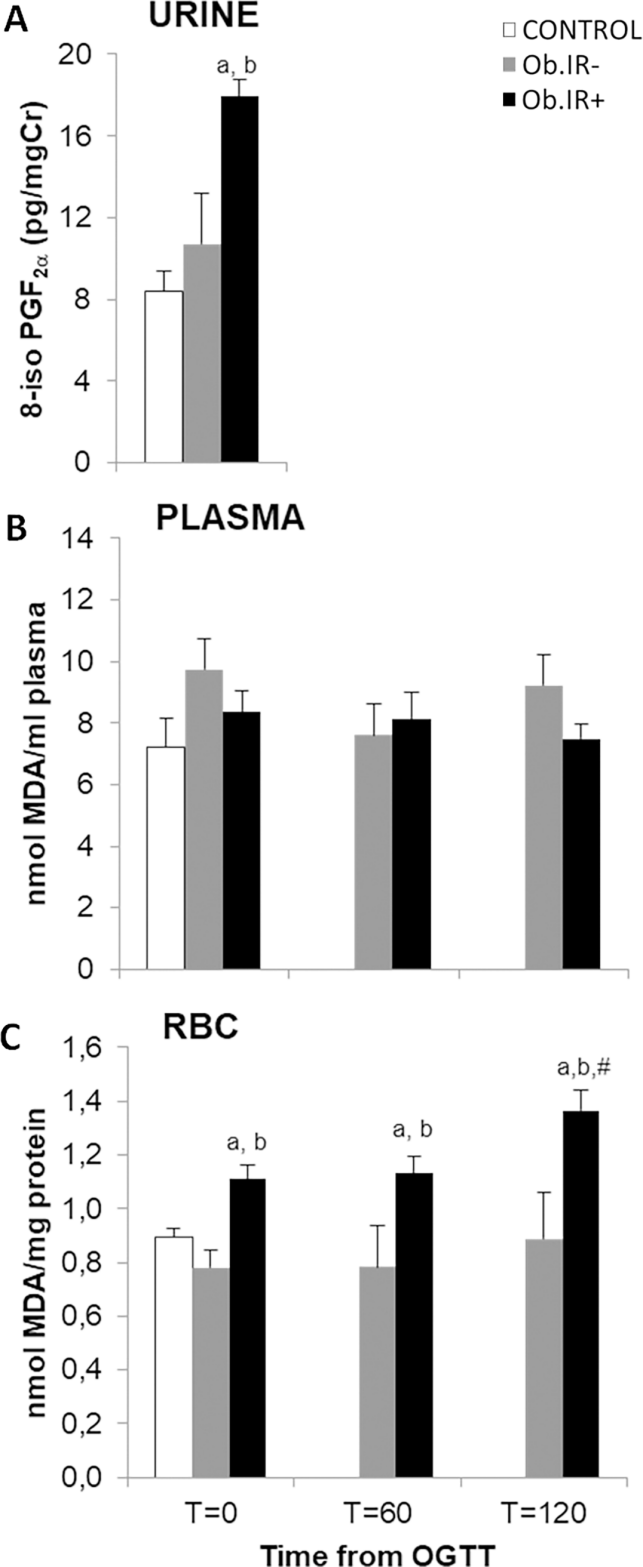
Lipoperoxide levels in urine, plasma and RBC of healthy and obese children. (A) Basal levels of urinary 8-isoprostane (8-iso PGF2α), (B) Plasmatic levels of TBARS at baseline and along 75 g. OGTT, and (C) RBC concentration of TBARS at baseline and along 75 g. OGTT. White bars represent the control group, grey bars represent Ob.IR- group, and black bars represent Ob.IR+ group. Data are represented as mean ± SEM. P<0.05 was considered for statistical significance. (a) show significant differences relative to control at baseline, (b) shows significant differences relative to Ob.IR-, and (#) intragroup differences along the OGTT respect to baseline values.

8-iso PGF_2α_ levels were analysed in urine samples, whilst TBARS levels were analysed in plasma and RBC as oxidative stress biomarkers (Fig 1A–1C). We found increased levels of 8-iso PGF_2α_ in the Ob.IR+ group as compared to controls and Ob.IR- groups. In plasma however, we found no differences in any of the variables analysed, while in RBC, results at base line (T = 0), were similar to urinary analysis, with increased TBARS levels in Ob.IR+ children as compared to the other two groups. Furthermore, OGTT induced increased TBARS in Ob-IR+ at the RBC fraction, with significant differences at T = 120’ with respect to baseline. These differences were not detected in plasma.

### Antioxidants markers in blood of healthy and obese children. Effects of OGTT in the obese groups

**Fig 2:**
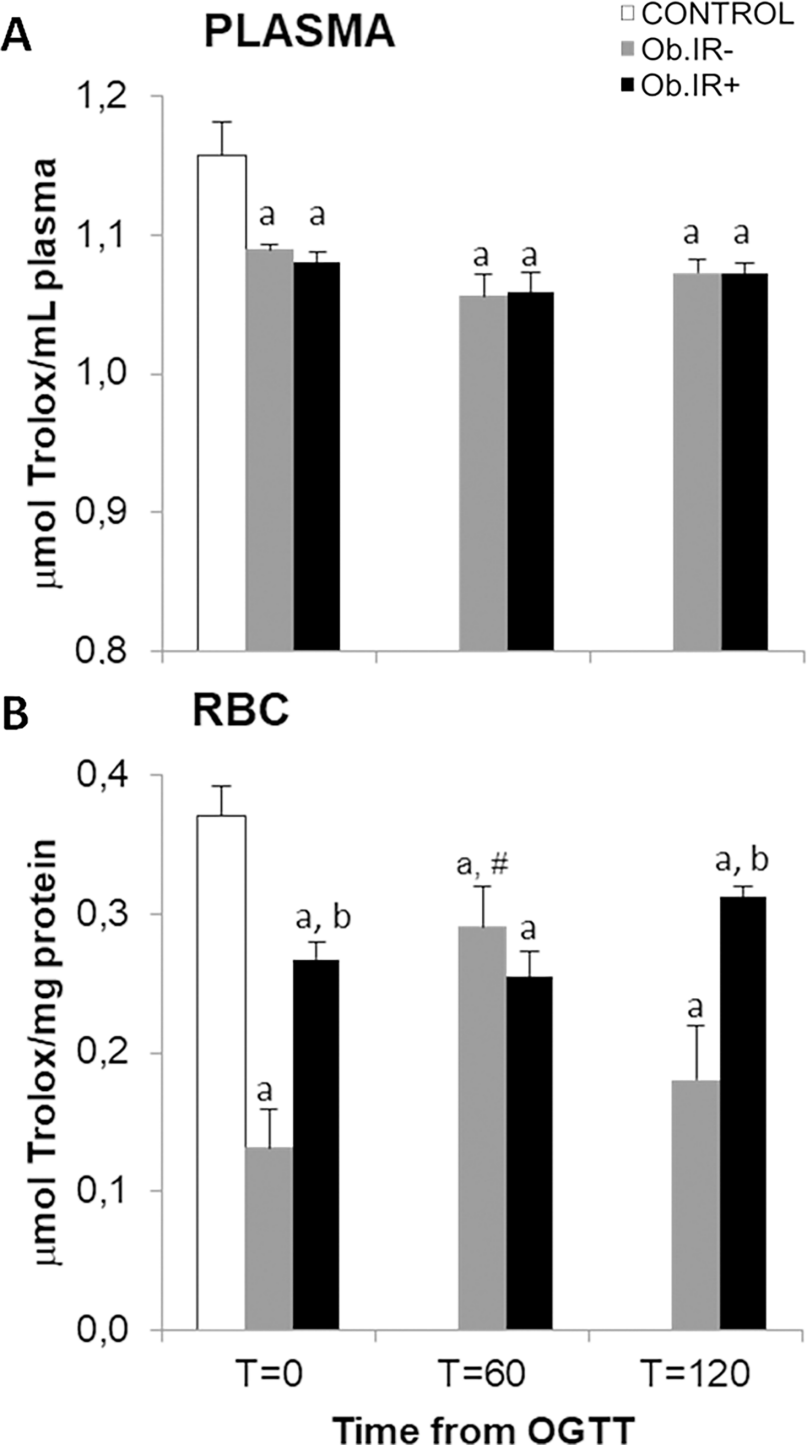
Total antoxiodant capacity (TAC) in venous blood in control and obese children. (A) Plasmatic TAC and, (B) RBC’s TAC at baseline and along 75 g. OGTT. White bars represent the control group, grey bars represent Ob.IR- group, and black bars represent Ob.IR+ group. Data are represented as mean ± SEM. P<0.05 was considered for statistical significance. (a) show significant differences relative to control at baseline, (b) shows significant differences relative to Ob.IR-, and (#) intragroup differences along the OGTT compared to baseline values.

Plasmatic total antioxidant capacity (TAC) in healthy and obese children after an overnight fast (T = 0), and along the OGTT in the obese groups, revealed that both obese groups had a significant decrease at baseline as compared to controls, but no differences between the obese groups, at any of the study points along the OGTT (Fig 2A). Again, data in RBC fraction differ from those in plasma. First, at baseline (T = 0), obese children showed reduced TAC in RBCs compared to healthy children, although, unexpectedly, Ob.IR- exhibited the lowest levels. Secondly, both obese groups behave differently along the OGTT; whilst the Ob.IR+ group remains stable along the glucose challenge, the Ob.IR- group showed a significant increase at T = 60, reaching Ob.IR+ levels, returning to their basal levels after 2 hours (Fig 2B).

**Fig 3:**
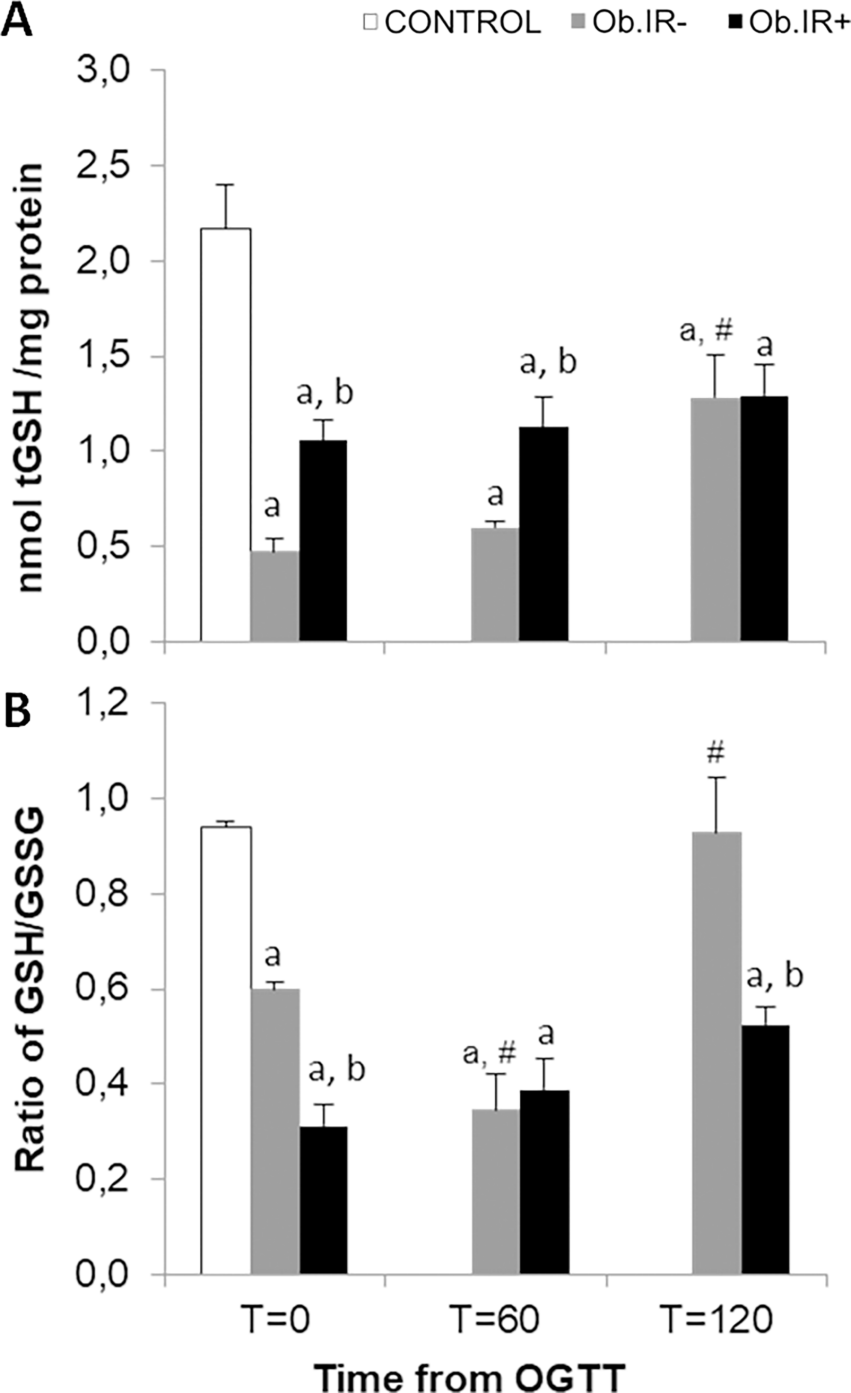
Redox status in RBC of healthy and obese children before and along an OGTT. (A) Total glutathione concentration (sum of all glutathione equivalents or tGSH, and (B) Reduced glutathione/oxidized glutathione rate, as measured in RBCs, at baseline and along the OGTT. White bars represent the control group, grey bars represent Ob.IR- group, and black bars represent Ob.IR+ group. Data are represented as mean ± SEM. P<0.05 was considered for statistical significance. (a) show significant differences relative to control at baseline, (b) shows significant differences relative to Ob.IR-, and (#) intragroup differences along the OGTT compared to baseline values.

In view of these results, it was considered relevant to analyse the concentration of total glutathione equivalents (tGSH) and redox status (GSH/GSSG ratio) in RBCs (Fig 3). Once more, we found a significant decrease in tGSH at baseline in both obese groups, when compared to controls, and again, the Ob.IR- group showed the lowest, and different behaviour along the curve. At T = 60’, there were still significant differences in tGSH between Ob.IR+ and Ob.IR-, but after 120 minutes the levels in the Ob.IR- group increased to match those of the Ob.IR+ group (Fig 3A).

When considering the GSH/GSSG ratio, we also found that controls exhibit a significantly higher ratio than either obese group, but in this case, the Ob.IR- group have a baseline level higher than the Ob.IR+ group, and the dynamics along the curve also differ; the Ob.IR+ group maintain a low level throughout the curve, while the Ob.IR- group experience a decrease at 60’, to levels as low as the Ob.IR+ group, but then recover to levels similar to controls at baseline (Fig 3B).

**Fig 4:**
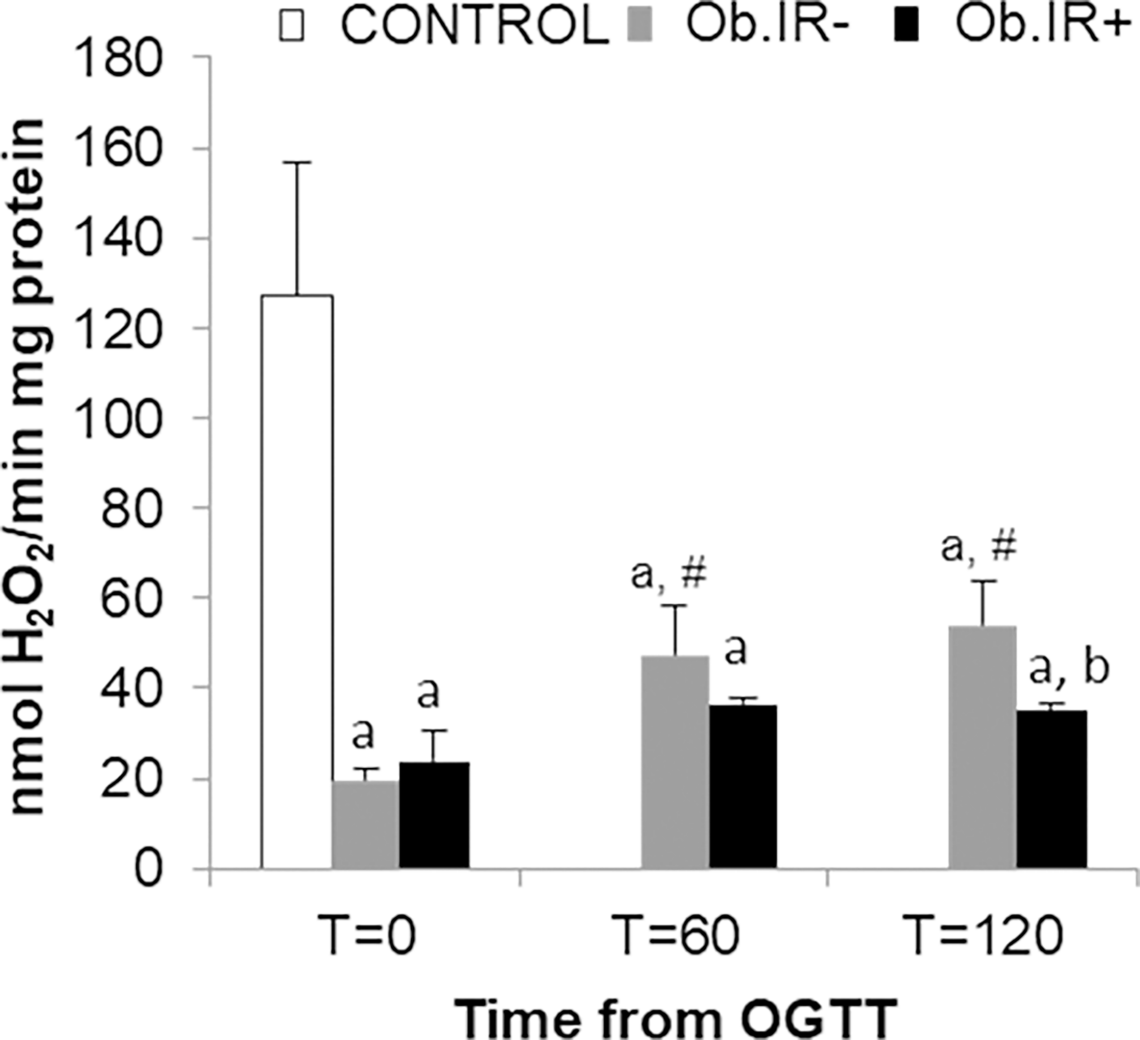
Catalase activity in RBC of healthy and obese children. At baseline and along the OGTT. Data is expressed in nmol H_2_O_2_ x min^-1^ x mg^-1^ of protein. White bars represent the control group, grey bars represent Ob.IR- group, and black bars represent Ob.IR+ group. Data are represented as mean ± SEM. P<0.05 was considered for statistical significance. (a) show significant differences relative to control at baseline, (b) shows significant differences relative to Ob.IR-, and (#) intragroup differences along the OGTT compared to baseline values.

Intraerythrocytary catalase activity (CAT) decreased significantly in both obese groups as compared to controls. Both (Ob.IR- and Ob.IR+), were found to have approximately a 16% of the CAT displayed by controls, but while the Ob.IR- group were able to increase their CAT along the OGTT, the Ob.IR+ group were unable to show such response, rendering a significant lower level at T = 120’ than the Ob.IR- group (Fig 4).

**Fig 5:**
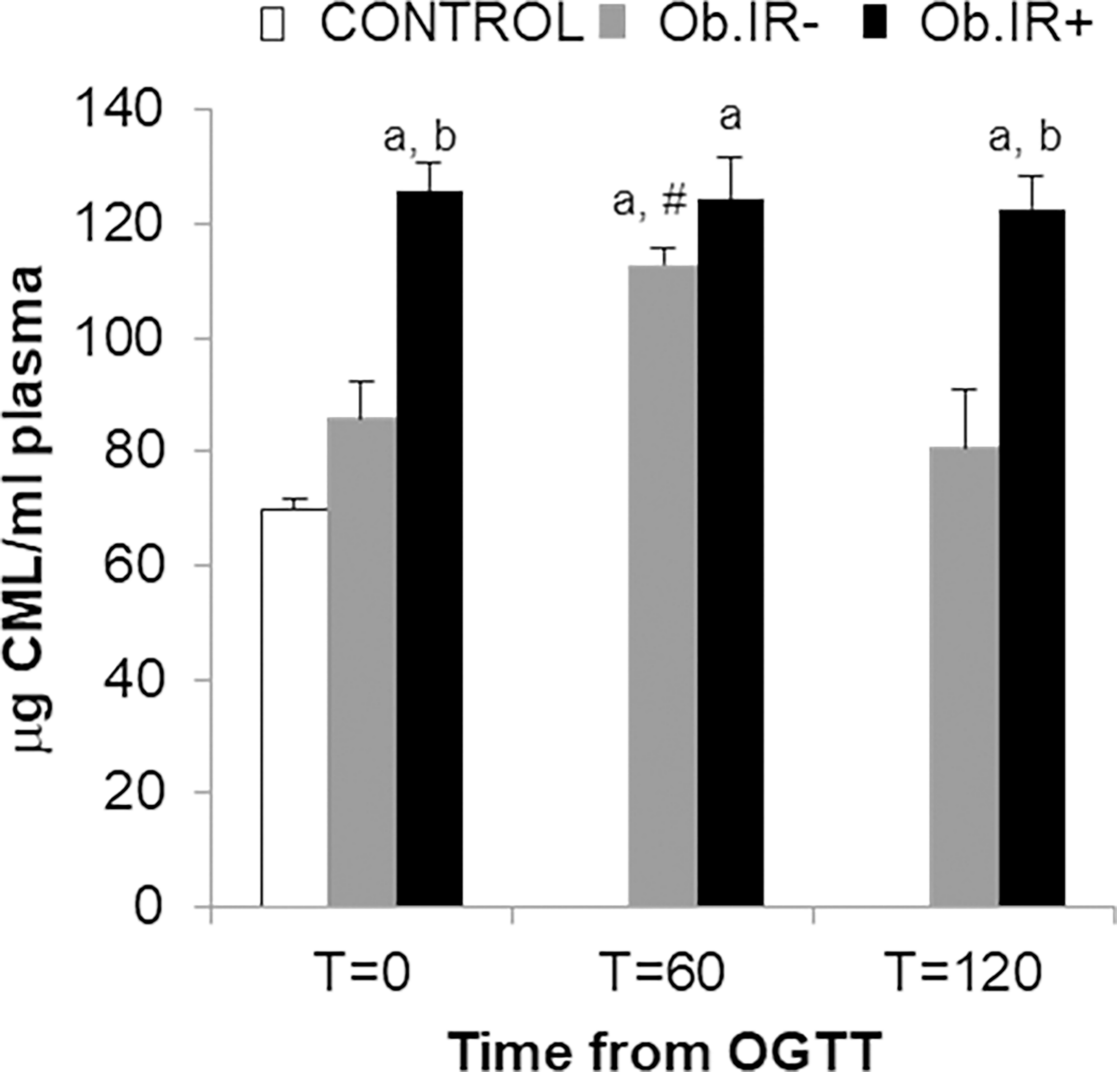
Plasmatic Advanced Glycation End Products (AGEs) in control and obese children. Measured at baseline and along the OGTT. Data is expressed in μg of CML/ml. White bars represent the control group, grey bars represent Ob.IR- group, and black bars represent Ob.IR+ group. Data are represented as mean ± SEM. P<0.05 was considered for statistical significance. (a) show significant differences relative to control at baseline, (b) shows significant differences relative to Ob.IR-, and (#) intragroup differences along the OGTT respect to baseline values.

We found levels of plasmatic Advanced Glycation End Products (AGEs) to behave in completely the opposite way to the GSH/GSSG ratio, all along the curve; thus, at baseline, both obese groups had significantly higher AGEs levels than controls, and the Ob.IR- group in turn, had lower levels than the Ob.IR+ group. Also, whilst the Ob.IR- group experience a significant increase at T = 60’, but eventually recovers to basal levels by T = 120’, the Ob.IR+ group show the highest levels all throughout the curve, with no significant variation (Fig 5).

### Correlation study

Finally, we explored the possible correlations among variables, including the biochemical data using Spearman’s Correlation Coefficient. We found an association between HOMA-IR and plasma AGEs concentration related to the levels of oxidative and antioxidant markers analysed at baseline. On the one hand, a significant positive correlation was found between HOMA-IR and lipoperoxides levels both, in urine and RBC, and also with plasma AGEs [0.605 (p = 0.010), 0.684 (p = 0.005) and 0.209 (p = 0.050), respectively]. On the contrary, a negative correlation was found between HOMA-IR and antioxidant activity of catalase and GSH/GSSG ratio [-0.446 (p = 0.001) and -0.689 (p = 0.002), respectively]. Finally, plasma AGEs concentrations were negatively associated to every variable related to antioxidant capacity (erythrocitary CAT, and tGSH, and plasmatic TAC) [-0.793 (p = 0.001), -0.673 (p = 0.006), -0.734 (p = 0.001) and -0.803 (p = 0.001), respectively] and redox status in venous blood [-0.710 (p = 0.001)].

## Discussion

Obesity is a worldwide health problem. It is a multi-factorial condition affecting both adults and children, with relevant metabolic complications that increase cardiovascular risk. However, according to the *International Diabetes Federation* guideline, metabolic syndrome cannot even be diagnosed in patients under the age of 10 (6). Nevertheless, the pathogenesis of such complications remains a matter of debate.

The combination of an excess production of ROS and RNS and a cellular inefficient antioxidant capacity, generates oxidative stress, which in turn is one of the most relevant molecular mechanisms underlying the development of obesity-induced metabolic complications.

We found that obese children with insulin resistance present impaired systemic oxidative status in contrast to healthy or obese children without insulin resistance. Notably, the Ob.IR+ group had a mean age of 9.27 years, thus most of them were under 10, and 8 out of the 12 were pre-pubescent. They presented higher levels of oxidative stress markers in RBCs and in urine and altered dynamics of their antioxidant response to OGTT. In addition, they had increased CRP and transaminases, suggesting some degree of underlying inflammation and non-alcoholic steatohepatitis, which have been previously associated with oxidative stress in obese children [2,26,27], and with an accelerated free fatty acid flux to portal circulation and serum AGEs accumulation in adults with metabolic syndrome [28,29]. Since puberty is a physiological risk factor for insulin resistance, and the Ob.IR- group has a higher proportion of pubertal patients, the findings of increased oxidative stress and blunted antioxidant response found in the Ob.IR+ group (in which there is a higher rate of prepubertal patients), strengthens the idea that these alterations happen in relation to the glucose metabolism status rather than in relation to age or pubertal status.

To the best of our knowledge, this is the first study showing the differences in oxidative stress and antioxidant markers in plasma in relation to intracellular fraction of RBCs in a group of obese children after an overnight fast, and in response to an OGTT. Our results at baseline (T = 0) found no differences in plasmatic lipoperoxides levels between both groups of obese children, while in urine and RBCs the Ob.IR+ group shows increased levels. Insulin resistance was positively correlated with AGEs basal levels in plasma, as reported in obese adults [30]. Thus, it is plausible that lipoperoxides levels in urine and RBC reflect the situation better than plasma. Our data is in accordance with the idea that oxidative stress induction is among the molecular mechanisms implicated in the development of metabolic complications in obese children, as has been previously proposed by others [31,32].

It is worth emphasizing here the role that excess of AGEs in plasma may be playing in the oxidative stress and inflammatory processes in the Ob.IR+ group. Our data supports the pivotal role of NADPH oxidase as the link between obesity, inflammation and oxidative stress [11,33]. Its overstimulation by plasmatic AGEs, which we found to be permanently increased in the Ob-IR+ group, could well be contributing to the consequent intracellular overproduction of ROS. This would explain the increased level of oxidative stress biomarkers in the erythrocytes of these patients, especially as the antioxidant systems of this group of patients have proven not to be efficient.

We also found differences in measuring the TAC in plasma versus RBCs. Both determinations rendered a higher TAC in controls versus both obese groups, but in RBCs we also found increased TAC in the group with insulin resistance as compared with the obese group with no metabolic complications. tGSH levels behave in a similar way, but in both groups were markedly lower than controls. Furthermore, the GSH/GSSG ratio was decreased in RBC of Ob.IR+ versus Ob.IR-, suggesting a higher depletion of GSH in the Ob.IR+ group as a result of their increased oxidative stress. Several chronic diseases which trigger increased production of ROS have been previously associated with erythrocyte GSH depletion; additionally, restoration of erythrocyte GSH concentration has positive therapeutic effects, while a low GSH concentration is a prognostic factor of a poor disease outcome [34,35]. However, catalase activity was similar in both obese groups, and again, both significantly lower than in controls, as previously reported by Ruperez *et al*. 2013 [16]. Furthermore, we also found a negative correlation [-0.446 (p = 0.001)] between insulin resistance and catalase activity in the obese groups.

Differences between obese groups in TAC and tGSH need to be discussed; finding lower levels of TAC and tGSH in obese children versus controls may reflect an exhaustion of antioxidant defences, and the reason for which the Ob.IR+ group show higher levels than Ob.IR-, when they have precisely increased TBARS and AGE levels, could be suggesting that obese children with insulin resistance exhibit ineffective defences against oxidation. Thus, even though they present higher antioxidant levels than the Ob.IR- group, these are insufficient to keep the balance with the oxidative stressors, resulting in increased lipoperoxide and AGE levels alongside a higher activation of the antioxidant mechanisms.

In this sense, the study of the dynamics performed along the OGTT provides additional information which may help explain the former findings. Lipoperoxide levels are constant throughout the curve in both groups of obese children: the Ob.IR- are able to keep lipoperoxide at the same levels that controls present at baseline, thus, we may infer that this group is managing to keep its lipoperoxides levels low, avoiding the oxidative stress induced by the acute glucose intake. The way they achieve this is by improving their antioxidant response, as demonstrated by the increased TAC at T = 60’, which will later return to baseline values. Moreover, they also display an increase in tGSH concentration and CAT activity along the OGTT. The GSH/GSSG ratio suffers a timely decrease in this Ob.IR- group (at T = 60’), showing a temporary depletion of GSH that is later recovered up to baseline values (similar to those in controls) by the end of the OGTT. Hence, our data shows how Ob.IR- children, even if at baseline they may show lower levels of antioxidants than controls, still have the ability to react to an acute stressor, mobilize their defences to achieve a lack of increase in lipoperoxide levels and an effective neutralization of the AGEs surge (that happens in the middle of the OGTT). In contrast, Ob.IR+ children, do not show any kind of response to the OGTT, and their TAC remain high but ineffective and lower than controls at baseline all along the curve. Additionally, their tGSH levels remain higher and unaltered by the OGTT, with a lower GSH/GSSG ratio, pointing to a GSH depletion as the result of a mismatch between GSH production and oxidation [36] and an inability to increase CAT with time. All these hyperactive defences are, however, unable to stop the increased lipoperoxide levels and the higher levels of AGEs all along the curve that was found in this group.

Taken together, our results suggest that obese children with insulin resistance suffer continuous oxidative stress and thus, their antioxidant mechanisms are close to depletion, overwhelmed and struggle to manage acute stressors. Meanwhile obese children who have not yet developed insulin resistance have a healthy response to acute stressors, allowing them not to increase their basal oxidation levels above the control group.

In conclusion, (i) Erythrocytes reflect changes in oxidative status earlier than plasma, and thus, we propose them as sensors of oxidative stress associated with insulin resistance in childhood obesity. (ii) Obese children without insulin resistance are able to effectively activate their defences in response to an acute stressor and avoid the oxidative damage, whereas obese children with insulin resistance demonstrate a constant increase in their antioxidant capacity in spite of increased oxidative stress when challenged. (iii) A depletion of RBCs’ reduced glutathione in obese children with insulin resistance is associated with a limited catalase activity.

This was a pilot study, with a limited sample size, and data should be interpreted with caution until reproduced. Further investigations are warranted to confirm these findings and to explore the underlying mechanisms.

## Supporting information

S1 Appendix Minimal anonymized data set. (XLSX)

S2 Appendix Method for red blood cell fraction purification. (TIF)
