# Supplementary figures and images for: Obesity induced alterations in redox homeostasis and oxidative stress are present from an early age
Source: PLoS One. 2018 Jan 25;13(1):e0191547. doi: 10.1371/journal.pone.0191547 (PMC5784965; doi:10.1371/journal.pone.0191547)

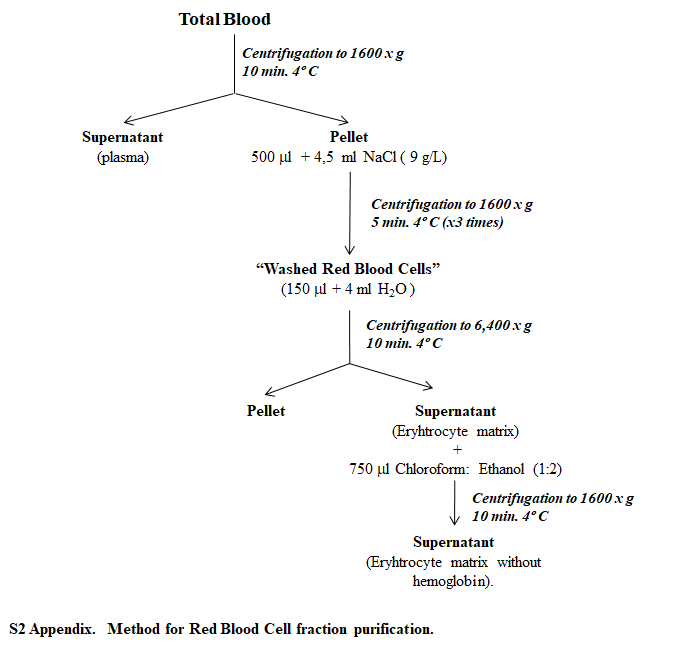

Supplement: S2 Appendix — (TIF) [file pone.0191547.s002.tif]
